# Invasive Pulmonary Aspergillosis in an Immunocompetent Patient: An Atypical Presentation

**DOI:** 10.7759/cureus.55469

**Published:** 2024-03-04

**Authors:** Aishwarya K Kedar, Babaji Ghewade, Ulhas Jadhav, Pankaj Wagh, Vivek D Alone

**Affiliations:** 1 Pulmonary Medicine, Jawaharlal Nehru Medical College, Datta Meghe Institute of Higher Education and Research, Wardha, IND

**Keywords:** clinical improvement, antifungal therapy, diagnosis, atypical presentation, immunocompetent, invasive pulmonary aspergillosis

## Abstract

Invasive pulmonary aspergillosis (IPA) is a severe fungal infection primarily affecting immunocompromised individuals. However, rare cases of IPA in immunocompetent patients have been reported, presenting diagnostic and therapeutic challenges. Here, we present a case of a 41-year-old immunocompetent male who presented with fever, cough with mucoid expectoration, and breathlessness. Despite the absence of traditional risk factors, imaging and laboratory findings led to the diagnosis of IPA. Prompt initiation of antifungal therapy resulted in clinical improvement. This case highlights the importance of considering IPA in the differential diagnosis of respiratory symptoms, even in immunocompetent individuals.

## Introduction

Invasive pulmonary aspergillosis (IPA) is a severe fungal infection primarily affecting immunocompromised individuals, such as those with hematologic malignancies, solid organ transplant recipients, or individuals undergoing immunosuppressive therapy [1]. However, rare instances of IPA have been reported in immunocompetent patients, posing diagnostic and therapeutic challenges due to atypical presentations and less obvious risk factors [2]. *Aspergillus* spp. is a ubiquitous fungus found in the environment, with *Aspergillus fumigatus* being the most common species implicated in IPA [3]. Inhalation of airborne conidia is the primary entry route, particularly in individuals with underlying lung disease or compromised immunity, leading to colonization and subsequent invasive disease [4].

The diagnosis of IPA in immunocompetent individuals requires a high index of suspicion, as clinical manifestations can vary widely and may mimic other respiratory conditions. Radiological findings, such as the "halo sign" on high-resolution computed tomography (HRCT), can aid in the diagnosis, although definitive confirmation often relies on microbiological and histopathological evaluation [5]. Early initiation of therapy is crucial to improve outcomes and reduce mortality rates associated with IPA, such as with antifungal therapy, primarily with triazoles such as voriconazole [1].

## Case presentation

A 41-year-old male patient presented with chief complaints of fever, cough with mucoid expectoration, and breathlessness for 15 days. He had no underlying comorbidities and was vitally stable throughout his evaluation. His systemic examination revealed bilateral vesicular breath sounds with no added adventitious sounds.

**Figure 1:**
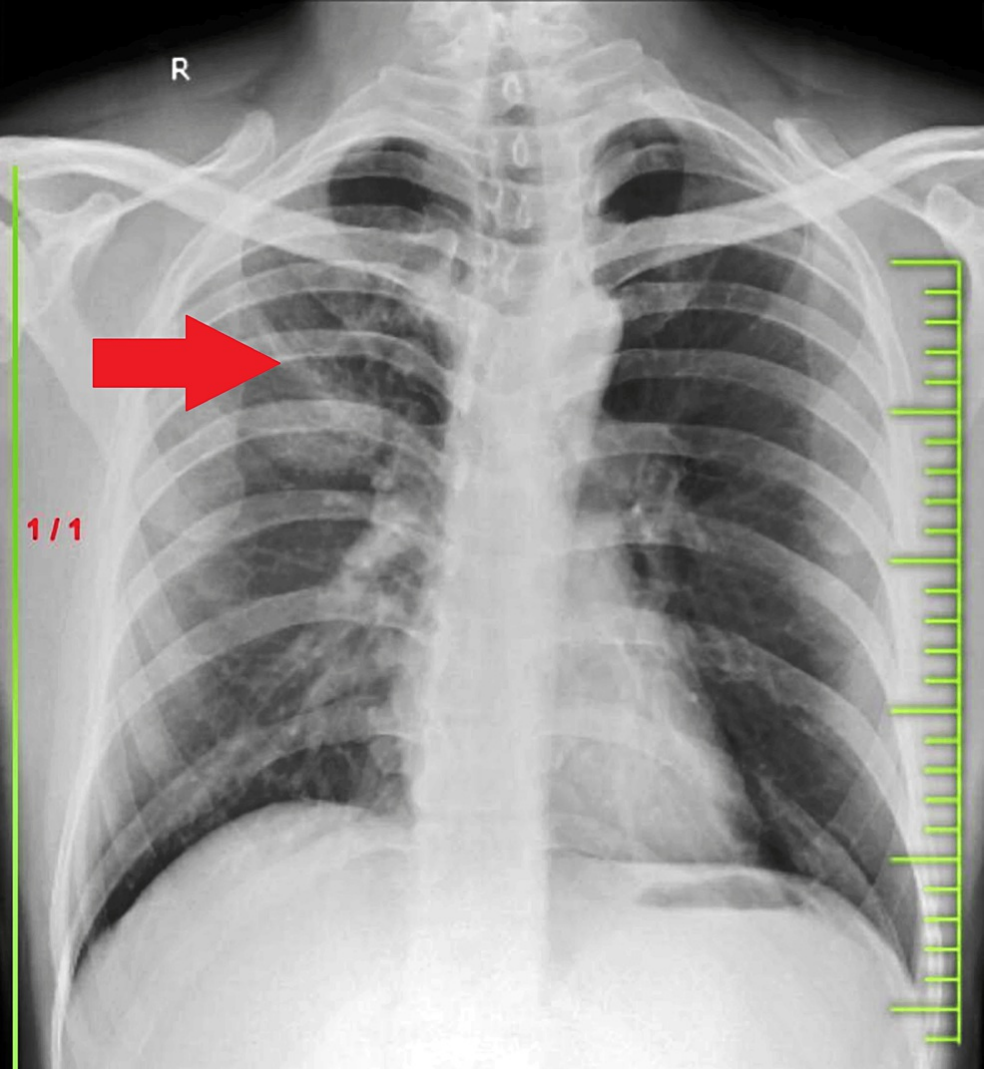
Chest radiography showing homogeneous rounded opacity occupying the right upper zone (posterior segment)

Chest radiography was done for the patient, which revealed a homogeneous rounded opacity occupying the right upper zone (posterior segment) (Figure 1). Subsequent sputum examination yielded negative results for acid-fast bacilli and nucleic acid amplification tests, microscopy, gram staining, and culture.

**Figure 2:**
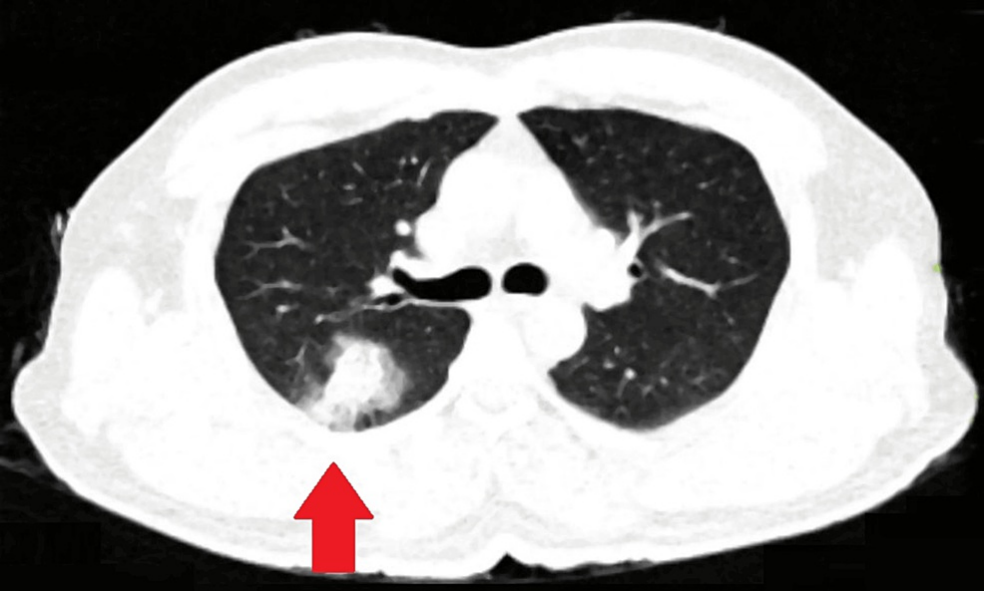
High-resolution computed tomography showing a well-defined consolidation measuring 2.6 × 2.2 × 2.4 cm with surrounding ground glass opacity in the superobasal segment of the lower lobe on the right side (the classical halo sign)

His blood investigations demonstrated a complete blood count of 12,000 cells/µL, with granulocytes accounting for 75% and lymphocytes for 30%. Liver and kidney function tests were within normal limits. Further evaluation via HRCT of the lungs revealed a well-defined consolidation measuring 2.6 × 2.2 × 2.4 cm, with surrounding ground-glass opacity in the posterior segment of the upper lobe and superobasal segment of the lower lobe of the right lung, indicative of the "halo sign" (Figure 2).

A bronchoscopy was planned, and a bronchoalveolar lavage (BAL) sample was collected for investigation. The BAL sample tested positive for galactomannan (*Aspergillus* antigen) via enzyme-linked immunosorbent assay (ELISA), with a concentration of 2.14 µg/L. Cytological examination of the BAL fluid confirmed the presence of *Aspergillus* spp. A diagnosis of invasive aspergillosis was made, and the patient was started on voriconazole. The patient's condition improved clinically, and he was discharged with instructions for a follow-up visit in 15 days.

## Discussion

IPA is a severe fungal infection primarily affecting immunocompromised individuals; however, rare cases in immunocompetent patients have been reported, posing diagnostic and therapeutic challenges [6]. Our case highlights the importance of considering IPA in the differential diagnosis of respiratory symptoms, even in patients without traditional risk factors. The atypical presentation of IPA in our immunocompetent patient highlights the need for a high index of suspicion, especially when faced with diagnostic ambiguity.

Imaging played a crucial role in our patient's diagnosis, with chest radiography revealing a rounded opacity in the right upper zone and HRCT demonstrating a consolidative lesion with surrounding ground-glass opacity, indicative of the "halo sign." These findings are consistent with previous reports of IPA in immunocompetent patients [7,8]. Laboratory investigations, including sputum examination and blood tests, were inconclusive initially. However, the detection of *Aspergillus* antigen (galactomannan) in the BAL fluid and cytological evidence of *Aspergillus* spp. facilitated the diagnosis of IPA. The galactomannan assay is a valuable tool in diagnosing IPA, particularly in immunocompromised patients [9].

Treatment of IPA typically involves antifungal therapy, with voriconazole being the preferred agent [10]. In our case, prompt initiation of voriconazole led to clinical improvement, highlighting the importance of early intervention in mitigating the morbidity and mortality associated with IPA. Regular follow-up is essential in monitoring treatment response and disease recurrence. Our patient demonstrated clinical improvement upon discharge, emphasizing the need for continued surveillance and adherence to therapy.

## Conclusions

In conclusion, our case highlights the occurrence of IPA in an immunocompetent patient presenting with fever, cough, and breathlessness. Despite the absence of traditional risk factors, including comorbidities, the diagnosis of IPA was established based on characteristic imaging findings, positive galactomannan assay, and cytological evidence of *Aspergillus* spp. in BAL fluid. Prompt initiation of voriconazole therapy resulted in clinical improvement, emphasizing the importance of early recognition and treatment in managing IPA. This case highlights the need for clinicians to maintain a high index of suspicion for fungal infections, including IPA, in patients presenting with respiratory symptoms, even in the absence of immunocompromised states. Further research is warranted to understand better the epidemiology, diagnostic approaches, and optimal management strategies for IPA in immunocompetent individuals. Regular follow-up is essential for monitoring treatment response and preventing disease recurrence. Continued surveillance and adherence to therapy are crucial in achieving favorable outcomes in patients with IPA. Overall, this case contributes to the growing literature on atypical presentations of IPA and emphasizes the importance of considering fungal infections in the differential diagnosis of respiratory illnesses.
